# Women as Doctors: Professor Hochenegg's Views on This Question

**Published:** 1913-07-26

**Authors:** 


### WOMEN AS DOCTORS.

### Professor Hochenegg's Views on this Question.

Under this head the *Times* reports that Professor Hochenegg, in the course of an interesting address to a meeting of the Austrian Women's Association, touched upon the question of the fitness of women for the medical profession. After pointing out that women are specially adapted for professional nursing, Professor Hochenegg proceeded to declare: "In my opinion, which I share with many of my colleagues, and which is increasingly confirmed the more I become acquainted with female medical students, women are not adapted for the work of a medical practitioner. This is not meant as censure, but only as a statement of fact. A doctor must judge independently and often act rapidly. His whole work demands qualities that are rarely found fully developed in the female sex. Despite the great industry and the high intelligence shown by many of my women students, they are destined to succumb in their struggle, or at least not to be able to attain a position corresponding to their work and to their sacrifice of the joys of life. . . . As nurses of the sick and the wounded, the conditions are entirely different. In this field women possess special power and fitness, and are not subject to male competition. Here their triumph is assured." This, of course, is a matter of opinion.

### SIR LAUDER BRUNTON'S REPLY TO PROFESSOR HOCHENEGG.

In connection with this declaration of the Austrian Professor, Sir Lauder Brunton writes to us under date July 21, 1913:—

"My attention has been called to an assertion by an Austrian doctor that although women are well suited for the calling of a nurse they are not fitted by intellect or temperament to be medical practitioners. To this I would reply:—

"(1) Many of us value the opinion of medical women in consultation about our patients.

"(2) That in 1910, when the British Medical Association held its annual meeting in London, a woman was elected as president of the Obstetric and Gynæcological Section.

"Although any one man might be mistaken as to the fitness of women to be consultants or officers of a great society, it is not possible that the whole body of practitioners in the United Kingdom could make a mistake in so important a matter."

[We shall be glad to open our columns to a full discussion of the question thus raised.]

